# Context-rich short answer questions (CR-SAQs) in assessment for learning in undergraduate medical education

**DOI:** 10.1080/10872981.2019.1674569

**Published:** 2019-10-01

**Authors:** Alistair Ludley

**Affiliations:** Imperial College London, School of Medicine, London, UK

Dear **editor**, the research article by Bird and colleagues [1] on the use of Context-Rich Short Answer Questions (CR-SAQs) in assessment *for* learning at medical school, was read with great interest and I thank the authors for their contribution. The study evaluated CR-SAQs as an assessment tool for learning, utilising data gathered from both medical students and faculty members to appraise the exam format. As a 5^th^-year medical student, I have been exposed to multiple question types including traditional formats such as Multiple-Choice Questions (MCQs) and Single Best Answer (SBAs), as well as more innovative Very Short Answer (VSA) and Clinical Prioritisation Questions (CPQs). Through this letter, I intend to appraise the original research and focus on the ramifications of CR-SAQs in Undergraduate Medical Education (UME).

The article reported that students changed their study habits in order to better prepare for CR-SAQ exams. This included how they studied harder, mastered the content and attained a deeper level of learning compared to how they would have prepared for MCQ exams. This could demonstrate that the CR-SAQ format has value as an assessment tool *for* learning. However, to substantiate this claim, it would be interesting to further investigate the subsequent long-term memory recall of the content examined by CR-SAQs compared to that examined by other formats such as by the MCQ. This could lend further evidence to fully understand the benefits of using CR-SAQs in examination *for* learning.

In order to reduce inter-rater variability between the marking of CR-SAQ responses, the authors had single faculty members grade all the answers for a given question in an exam paper. Whilst this would ensure all students are graded in the same fashion, this method may have failed to remove any potential systematic examiner bias. To alleviate such bias, the authors could have recruited another set of independent examiners to mark the same set of questions, with an average mark then generated [2]. Alternatively, a sample of examination papers could have been scrutinised by an external examiner.

The use of CR-SAQs seem to have a place in UME, but their use should be supplemented with other, well-researched and established question formats, such as the MCQ and SBA. With appropriate supplementation, multiple domains of learning can be examined [3], from factual recall in SBAs to clinical prioritisation in CPQs. As an assessment tool *for* learning, it is important that more research is done into CR-SAQs with a specific focus on their ability to impact long-term memory recall.
